# Transcriptional Changes Common to Human Cocaine, Cannabis and Phencyclidine Abuse

**DOI:** 10.1371/journal.pone.0000114

**Published:** 2006-12-27

**Authors:** Elin Lehrmann, Carlo Colantuoni, Amy Deep-Soboslay, Kevin G. Becker, Ross Lowe, Marilyn A. Huestis, Thomas M. Hyde, Joel E. Kleinman, William J. Freed

**Affiliations:** 1 Cellular Neurobiology Research Branch, National Institute on Drug Abuse (NIDA) Intramural Research Program, National Institutes of Health, Department of Health and Human Services, Baltimore, Maryland, United States of America; 2 Clinical Brain Disorders Branch, GCAP, National Institute of Mental Health (NIMH) Intramural Research Program, National Institutes of Health, Department of Health and Human Services, Bethesda, Maryland, United States of America; 3 Research Resources Branch, National Institute on Aging (NIA) Intramural Research Program, National Institutes of Health, Department of Health and Human Services, Baltimore, Maryland, United States of America; 4 Chemistry and Drug Metabolism Section, National Institute on Drug Abuse (NIDA) Intramural Research Program, National Institutes of Health, Department of Health and Human Services, Baltimore, Maryland, United States of America; James Cook University, Australia

## Abstract

A major goal of drug abuse research is to identify and understand drug-induced changes in brain function that are common to many or all drugs of abuse. As these may underlie drug dependence and addiction, the purpose of the present study was to examine if different drugs of abuse effect changes in gene expression that converge in common molecular pathways. Microarray analysis was employed to assay brain gene expression in postmortem anterior prefrontal cortex (aPFC) from 42 human cocaine, cannabis and/or phencyclidine abuse cases and 30 control cases, which were characterized by toxicology and drug abuse history. Common transcriptional changes were demonstrated for a majority of drug abuse cases (N = 34), representing a number of consistently changed functional classes: Calmodulin-related transcripts (CALM1, CALM2, CAMK2B) were decreased, while transcripts related to cholesterol biosynthesis and trafficking (FDFT1, APOL2, SCARB1), and Golgi/endoplasmic reticulum (ER) functions (SEMA3B, GCC1) were all increased. Quantitative PCR validated decreases in calmodulin 2 (CALM2) mRNA and increases in apolipoprotein L, 2 (APOL2) and semaphorin 3B (SEMA3B) mRNA for individual cases. A comparison between control cases with and without cardiovascular disease and elevated body mass index indicated that these changes were not due to general cellular and metabolic stress, but appeared specific to the use of drugs. Therefore, humans who abused cocaine, cannabis and/or phencyclidine share a decrease in transcription of calmodulin-related genes and increased transcription related to lipid/cholesterol and Golgi/ER function. These changes represent common molecular features of drug abuse, which may underlie changes in synaptic function and plasticity that could have important ramifications for decision-making capabilities in drug abusers.

## Introduction

While human drug abusers exhibit specific preferences in their individual drugs-of-choice, polysubstance abuse is the rule, not the exception [1]. Animal studies have suggested that although different drugs of abuse have unique and specific mechanisms of action, the same molecular pathways may be involved in mediating common functional effects of multiple drugs of abuse [2]. These molecular pathways may therefore reflect common changes in brain function that promote continued drug use and compulsive drug-seeking behavior, irrespective of which particular drugs are abused.

Multiple brain regions are involved in the establishment and maintenance of addictive behavior. The prefrontal cortical regulation of cognitive and emotional processes is changed by drug abuse, such that inhibitory control of these processes is deficient and drug use is reinforced [3], [4]. The aPFC, defined as the anterior pole of Brodmann Area 10 (BA10), contains fewer cells, but with a higher spine density and length, than any other cortical region [5]. It is reciprocally connected to the prefrontal anterior temporal and cingulate cortices and has been suggested to serve an important integrative role in the pursuit of behavioral goals [6]. Concurrent activation of the aPFC and the orbitofrontal cortex has been demonstrated following administration of cocaine to cocaine-abusing individuals [7]. As a consequence, altered function of the aPFC could have important ramifications for decision-making capability in drug abusers.

Microarray analysis has been used to identify transcriptional changes in human neuropsychiatric disorders, including schizophrenia, depression and bipolar disorder [8]–[12] and in association with specific abused drugs [13]–[16]. Missing from these studies, however, are microarray analyses of the general transcriptional features of drug abuse *per se*. To address this topic, we performed a microarray study of human postmortem aPFC from 30 control cases and 42 drug abuse cases with varied drug abuse histories. A series of cases with cocaine, cannabis and/or phencyclidine as the primary drugs of abuse were examined for common patterns of regulation of gene expression that would represent identifiable biological functions. By classifying consistently regulated transcripts into biologically-relevant functional groups, we identified decreased expression of transcripts involved in calmodulin-related signaling and increased expression of transcripts involved in lipid/cholesterol metabolism and Golgi/ER-related function as being common molecular features involved in multiple patterns of human drug abuse.

## Methods

### Source and selection of cases

A series of 646 consecutive cases from the brain repository at the Clinical Brain Disorders Branch (NIMH IRP) were reviewed for evidence of drug abuse by either history or toxicology. Of the 138 cases identified, 50 cases were identified by a history of drug abuse, 25 cases by a positive toxicology test, and 63 cases were identified by both measures. Cases were excluded on the basis of co-morbid neurological or major psychiatric disorders, abnormal microscopic or macroscopic neuropathology, poor RNA quality, postmortem intervals (PMI) >72 hrs, or brain pH<6.0. A subset of 42 cases was retained, for which clinical case histories were compiled from chart records, medical examiner's files and structured interviews with next-of-kin to the extent possible. Controls (N = 30) were similarly selected from a well-characterized control cohort [17]. To limit the impact of confounds from any one control, each drug abuse case was matched to four controls that both individually and on average were the best matches for brain pH, PMI, age, gender, ethnicity and smoking history. Group mean demographic data are provided for drug abuse and control cases in *Table 1*, which demonstrate that brain pH, PMI, age and gender did not differ significantly between drug abusers and their individual control tetrads. Demographic data for individual cases are provided in Table S1.

**Table 1:**
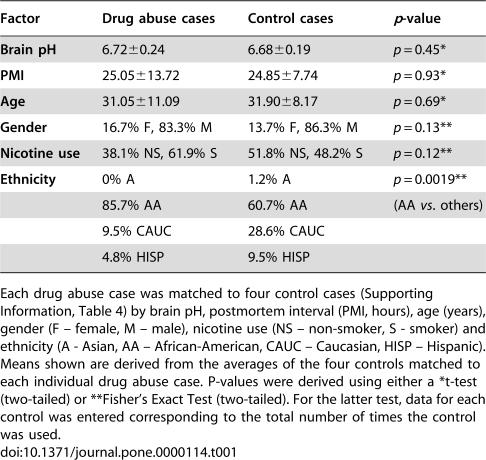
Summary of the demographic information for drug abuse (42) and control cases (30).

Factor	Drug abuse cases	Control cases	*p*-value
**Brain pH**	6.72±0.24	6.68±0.19	*p* = 0.45*
**PMI**	25.05±13.72	24.85±7.74	*p* = 0.93*
**Age**	31.05±11.09	31.90±8.17	*p* = 0.69*
**Gender**	16.7% F, 83.3% M	13.7% F, 86.3% M	*p* = 0.13**
**Nicotine use**	38.1% NS, 61.9% S	51.8% NS, 48.2% S	*p* = 0.12**
**Ethnicity**	0% A	1.2% A	*p* = 0.0019**
	85.7% AA	60.7% AA	(AA *vs*. others)
	9.5% CAUC	28.6% CAUC	
	4.8% HISP	9.5% HISP	

Each drug abuse case was matched to four control cases (Supporting Information, Table 4) by brain pH, postmortem interval (PMI, hours), age (years), gender (F – female, M – male), nicotine use (NS – non-smoker, S - smoker) and ethnicity (A - Asian, AA – African-American, CAUC – Caucasian, HISP – Hispanic). Means shown are derived from the averages of the four controls matched to each individual drug abuse case. P-values were derived using either a *t-test (two-tailed) or **Fisher's Exact Test (two-tailed). For the latter test, data for each control was entered corresponding to the total number of times the control was used.

### Toxicological evaluation

General toxicological information on drugs of abuse present at death was obtained from the medical examiner's office, and supplemented with additional toxicological testing of blood or brain to validate and extend the scope of drugs initially tested (National Medical Services, Willow Grove, PA). Gas chromatography-mass spectrometry (GC-MS) was employed to examine cerebellar tissue from all 72 cases for the presence of cocaine, amphetamine, phencyclidine, opioids and their metabolites [18]. In addition to this general toxicological evaluation, hair toxicology was employed for 31 drug abuse cases and 10 control cases, which provided a retrospective evaluation of exposure to cocaine, phencyclidine, amphetamines, opioids and cannabinoids (Psychemedics Corporation, Culver City, CA) in the months prior to death. General and hair toxicological findings are summarized in *Table 2*.

**Table 2:**
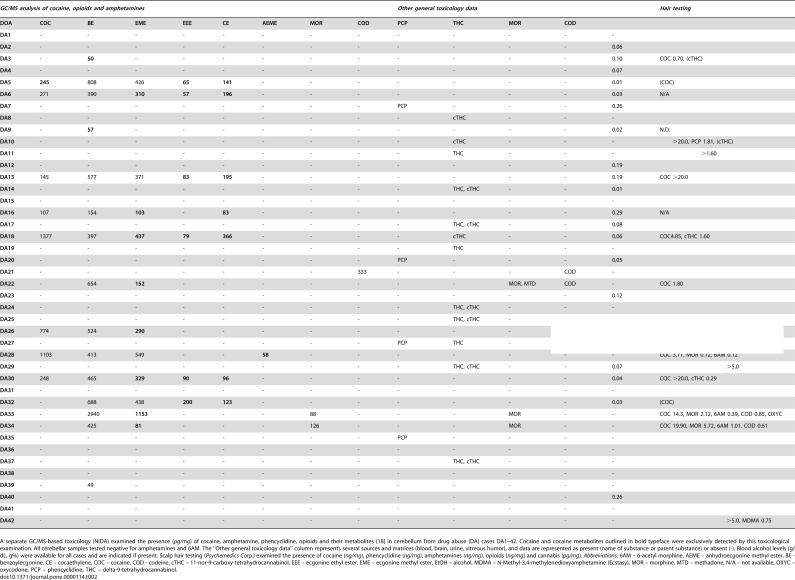
Toxicological evaluation.

*GC/MS analysis of cocaine, opioids and amphetamines*									*Other general toxicology data*					*Hair testing*
**DOA**	**COC**	**BE**	**EME**	**EEE**	**CE**	**AEME**	**MOR**	**COD**	**PCP**	**THC**	**MOR**	**COD**	**EtOH**	**COC, PCP, cTHC, OPIOIDS, AMPHETAMINES**
**DA1**	-	-	-	-	-	-	-	-	-	-	-	-	-	cTHC 1.50
**DA2**	-	-	-	-	-	-	-	-	-	-	-	-	0.06	COC 1.77, cTHC 0.49
**DA3**	-	**50**	-	-	-	-	-	-	-	-	-	-	0.10	COC 0.70, (cTHC)
**DA4**	-	-	-	-	-	-	-	-	-	-	-	-	0.07	(COC)
**DA5**	**245**	808	426	**65**	**141**	-	-	-	-	-	-	-	0.01	(COC)
**DA6**	271	390	**310**	**57**	**196**	-	-	-	-	-	-	-	0.03	N/A
**DA7**	-	-	-	-	-	-	-	-	PCP	-	-	-	0.26	N/A
**DA8**	-	-	-	-	-	-	-	-	-	cTHC	-	-	-	COC 0.58, cTHC 3.19
**DA9**	-	**57**	-	-	-	-	-	-	-	-	-	-	0.02	N.D.
**DA10**	-	-	-	-	-	-	-	-	-	cTHC	-	-	-	COC >20.0, PCP 1.81, (cTHC)
**DA11**	-	-	-	-	-	-	-	-	-	THC	-	-	-	COC 0.81, cTHC >1.60
**DA12**	-	-	-	-	-	-	-	-	-	-	-	-	0.19	N/A
**DA13**	145	577	371	**83**	**195**	-	-	-	-	-	-	-	0.19	COC >20.0
**DA14**	-	-	-	-	-	-	-	-	-	THC, cTHC	-	-	0.01	N/A
**DA15**	-	-	-	-	-	-	-	-	-	-	-	-	-	N/A
**DA16**	107	154	**103**	-	**83**	-	-	-	-	-	-	-	0.29	N/A
**DA17**	-	-	-	-	-	-	-	-	-	THC, cTHC	-	-	0.08	cTHC 4.10
**DA18**	1377	397	**437**	**79**	**366**	-	-	-	-	cTHC	-	-	0.06	COC4.85, cTHC 1.60
**DA19**	-	-	-	-	-	-	-	-	-	THC	-	-	-	N/A
**DA20**	-	-	-	-	-	-	-	-	PCP	-	-	-	0.05	N/A
**DA21**	-	-	-	-	-	-	-	333	-	-	-	COD	-	N/A
**DA22**	-	654	**152**	-	-	-	-	-	-	-	MOR, MTD	COD	-	COC 1.80
**DA23**	-	-	-	-	-	-	-	-	-	-	-	-	0.12	COC 0.45
**DA24**	-	-	-	-	-	-	-	-	-	THC, cTHC	-	-	-	COC 0.03, cTHC 0.42
**DA25**	-	-	-	-	-	-	-	-	-	THC, cTHC	-	-	-	COC 8.04, PCP 0.32, cTHC 2.55
**DA26**	774	524	**290**	-	-	-	-	-	-	-	-	-	-	N/A
**DA27**	-	-	-	-	-	-	-	-	PCP	THC	-	-	-	COC 3.53, PCP 2.94, cTHC 0.64
**DA28**	1103	413	549	-	-	**58**	-	-	-	-	-	-	-	COC 3.71, MOR 0.12, 6AM 0.12
**DA29**	-	-	-	-	-	-	-	-	-	THC, cTHC	-	-	0.07	COC 7.18, PCP 1.53, cTHC >5.0
**DA30**	248	465	**329**	**90**	**96**	-	-	-	-	-	-	-	0.04	COC >20.0, cTHC 0.29
**DA31**	-	-	-	-	-	-	-	-	-	-	-	-	-	COC 9.39, cTHC 1.12
**DA32**	-	688	438	**200**	**123**	-	-	-	-	-	-	-	0.03	(COC)
**DA33**	-	2940	**1153**	-	-	-	88	-	-	-	MOR	-	-	COC 14.3, MOR 2.12, 6AM 0.39, COD 0.85, OXYC 0.07
**DA34**	-	425	**81**	-	-	-	126	-	-	-	MOR	-	-	COC 19.90, MOR 5.72, 6AM 1.01, COD 0.61
**DA35**	-	-	-	-	-	-	-	-	PCP	-	-	-	-	cTHC 0.94
**DA36**	-	-	-	-	-	-	-	-	-	-	-	-	-	COC 0.56, MOR 0.06, 6AM 0.45
**DA37**	-	-	-	-	-	-	-	-	-	THC, cTHC	-	-	-	COC 1.23, cTHC 2.15
**DA38**	-	-	-	-	-	-	-	-	-	-	-	-	-	cTHC 1.02
**DA39**	-	49	-	-	-	-	-	-	-	-	-	-	-	N/A
**DA40**	-	-	-	-	-	-	-	-	-	-	-	-	0.26	NEG
**DA41**	-	-	-	-	-	-	-	-	-	-	-	-	-	(OPIOIDS)
**DA42**	-	-	-	-	-	-	-	-	-	-	-	-	-	COC 1.23, PCP 0.15, cTHC >5.0, MDMA 0.75

A separate GC/MS-based toxicology (NIDA) examined the presence (*pg/mg*) of cocaine, amphetamine, phencyclidine, opioids and their metabolites [18] in cerebellum from drug abuse (DA) cases DA1–42. Cocaine and cocaine metabolites outlined in bold typeface were exclusively detected by this toxicological examination. All cerebellar samples tested negative for amphetamines and 6AM. The “Other general toxicology data” column represents several sources and matrices (blood, brain, urine, vitreous humor), and data are represented as present (name of substance or parent substance) or absent (-). Blood alcohol levels (g/dL, g%) were available for all cases and are indicated if present. Scalp hair testing (*Psychemedics Corp*.) examined the presence of cocaine (*ng/mg*), phencyclidine (*ng/mg*), amphetamines (*ng/mg*), opioids (*ng/mg*) and cannabis (*pg/mg*). *Abbreviations*: 6AM – 6-acetyl morphine, AEME – anhydroecgonine methyl ester, BE - benzoylecgonine, CE – cocaethylene, COC – cocaine, COD - codeine, cTHC – 11-nor-9-carboxy-tetrahydrocannabinol, EEE – ecgonine ethyl ester, EME – ecgonine methyl ester, EtOH – alcohol, MDMA – N-Methyl-3,4-methylenedioxyamphetamine (Ecstasy), MOR – morphine, MTD – methadone, N/A – not available, OXYC – oxycodone, PCP – phencyclidine, THC – delta-9-tetrahydrocannabinol.

### Microarray experiments

Microarray experiments were essentially carried out as previously described [14]. Briefly, RNA was extracted from pulverized gray matter from BA10 (dorsal aspect, frontal pole) using Trizol reagent (Invitrogen, Carlsbad, CA). The quality and quantity of sample RNA was evaluated by Bioanalyzer electrophoresis (Agilent, Palo Alto, CA). Eight µg total RNA from each sample was reverse transcribed into ^33^P-labeled individual cDNA samples, and divided for duplicate hybridizations to Mammalian Gene Collection (MGC) arrays containing 9216 cDNA clones from the MGC clone set [19]. The arrays were exposed to a low-energy phosphor screen (Molecular Dynamics, Sunnyvale, CA) for 5 days, the screen scanned (Phosphorimager 860, Molecular Dynamics), and pixel intensities quantified using ImageQuant (Molecular Dynamics).

### Z-score transformation

The microarray data sets were analyzed using the *z*-score transformation normalization method [20], in which log-transformed and normalized hybridization intensity values provided the basis for *p*-value-based significance calculations by a *z*-test. Two-tailed *p*-values were used to identify transcripts with decreased or increased expression, respectively. Individual *z*-ratio data represent a normalized ratio between experimental and control cases.

### Hierarchical clustering

Hierarchical clustering of *z*-ratio data using Genesis software [21] was used to assess the overall relatedness of the global transcriptional changes for all drug abuse cases.

### Groups defined by global transcriptional profiles: selection and GAP criteria

**Figure 1:**
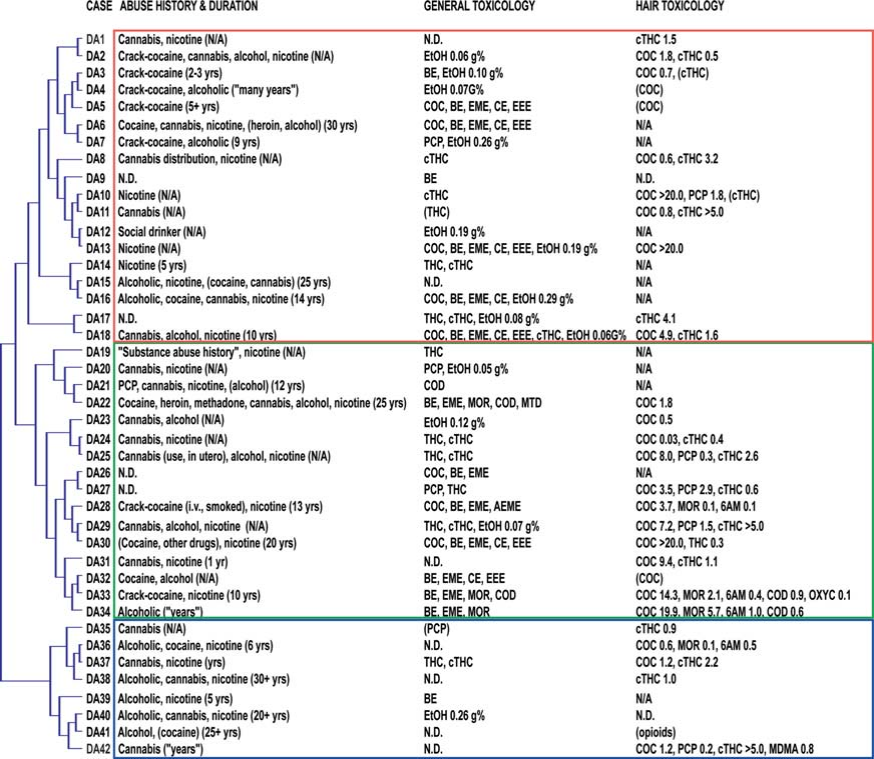
Hierarchical clustering identified three main groups of drug abuse cases. Hierarchical clustering of individual transcriptional profiles from comparisons of drug abuse cases and their individual four best-matched controls identified three main groups of drug abusers: Group I (DA1–18), Group II (DA19–34) and Group III (DA35–42). A summary of toxicology and drug abuse history for each case in the clustering dendrogram indicated cocaine use in a majority of cases, while presence of alcohol in Group I, and opioids and phencyclidine in Group II might underlie differences in Group I and II individuals. Group III cases differed markedly from other cases, which may be related to the absence or low levels of abused drug in most cases, a history of alcohol dependence, or to underlying medical conditions. Insufficient specimen for quantitative analysis of a positive hair test screening is indicated by a parenthesis around the substance name, units are ng/mg, except for cTHC (pg/mg). Abbreviations: 6AM – 6-acetyl morphine, AEME – anhydroecgonine methyl ester, BE - benzoylecgonine, CE – cocaethylene, COC – cocaine, COD - codeine, cTHC – 11-nor-9-carboxy-tetrahydrocannabinol, EEE – ecgonine ethyl ester, EME – ecgonine methyl ester, EtOH – alcohol, g% - g/dL, MDMA – N-Methyl-3,4-methylenedioxyamphetamine (Ecstasy), MOR – morphine, MTD – methadone, N/A – not available, OXYC – oxycodone, PCP – phencyclidine, THC – delta-9-tetrahydrocannabinol.

Two-tailed *p*-values for individual transcripts were averaged within each of the three groups, and formed the basis for selection of transcripts by group average *p*-value (GAP) criteria. Transcripts for which one (or more) GAP was ≤0.01 (or ≥0.99) were included to select transcripts strongly decreased (or increased, respectively) in individual groups, while transcripts where one (or more) GAP ≤0.05 (or ≥0.95) and one (or more) GAP ≤0.10 (or ≥0.90) were selected to include transcripts common to any two (or more) groups. Cases DA 17–18 were omitted from Group I GAP for this calculation only as they represented an intermediate between Group I and Group II (*Fig. 1*).

### Groups defined by toxicology: selection and GAP criteria

General toxicology (all toxicological data except hair testing) detected drugs of abuse in 26 Group I–II cases. Two cases with unknown drug abuse histories and negative or unavailable hair toxicological tests were excluded. The remaining 24 cases were divided among 12 cocaine (COC+), 9 cannabis (THC+), and 3 phencyclidine (PCP+) cases. The probability of decreased or increased expression were selected as GAP ≤0.10 and ≥0.90, respectively.

To select transcripts which were similarly changed in the groups defined by toxicology described above and also in all 34 Group I–II cases, transcripts with average *p*-values ≤0.10 (or ≥0.90) for all cases were selected if (*i*) at least 30 cases (≈90%) had GAP scores ≤0.10 (or ≥0.90), and (*ii*) GAP ≤0.06 (or ≥0.94) for two (or three) of the groups defined by general toxicology.

### Specificity of the findings

#### Ethnicity

GAP scores for six African-American male (AAM) drug abuse cases (DA3, 4, 5, 14, 20, 26) which each were matched exclusively to four AAM control cases were employed to examine if differences in ethnicity played a significant factor in transcript selection. AAM GAP scores are included in Table 3.

**Table 3:**
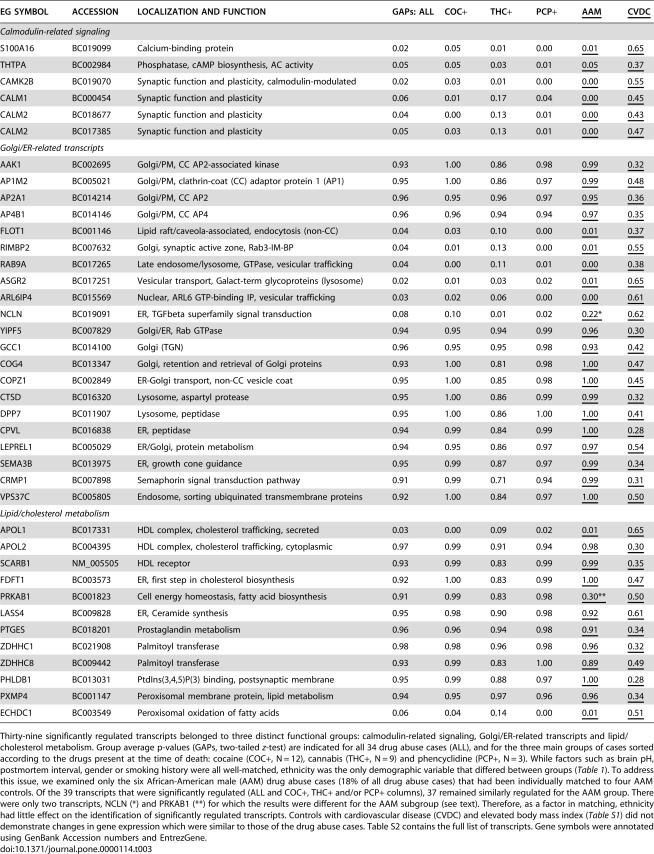
Significantly regulated transcripts identify three functional groups as shared across cocaine, cannabis and phencyclidine abuse cases.

EG SYMBOL	ACCESSION	LOCALIZATION AND FUNCTION	GAPs: ALL	COC+	THC+	PCP+	AAM	CVDC
*Calmodulin-related signaling*								
S100A16	BC019099	Calcium-binding protein	0.02	0.05	0.01	0.00	0.01	0.65
THTPA	BC002984	Phosphatase, cAMP biosynthesis, AC activity	0.05	0.05	0.03	0.01	0.05	0.37
CAMK2B	BC019070	Synaptic function and plasticity, calmodulin-modulated	0.02	0.03	0.01	0.00	0.00	0.55
CALM1	BC000454	Synaptic function and plasticity	0.06	0.01	0.17	0.04	0.00	0.45
CALM2	BC018677	Synaptic function and plasticity	0.04	0.00	0.13	0.01	0.00	0.43
CALM2	BC017385	Synaptic function and plasticity	0.05	0.03	0.13	0.01	0.00	0.47
*Golgi/ER-related transcripts*								
AAK1	BC002695	Golgi/PM, CC AP2-associated kinase	0.93	1.00	0.86	0.98	0.99	0.32
AP1M2	BC005021	Golgi/PM, clathrin-coat (CC) adaptor protein 1 (AP1)	0.95	1.00	0.86	0.97	0.99	0.48
AP2A1	BC014214	Golgi/PM, CC AP2	0.96	0.95	0.96	0.97	0.95	0.36
AP4B1	BC014146	Golgi/PM, CC AP4	0.96	0.96	0.94	0.94	0.97	0.35
FLOT1	BC001146	Lipid raft/caveola-associated, endocytosis (non-CC)	0.04	0.03	0.10	0.00	0.01	0.37
RIMBP2	BC007632	Golgi, synaptic active zone, Rab3-IM-BP	0.04	0.01	0.13	0.00	0.01	0.55
RAB9A	BC017265	Late endosome/lysosome, GTPase, vesicular trafficking	0.04	0.00	0.11	0.01	0.00	0.38
ASGR2	BC017251	Vesicular transport, Galact-term glycoproteins (lysosome)	0.02	0.01	0.03	0.02	0.01	0.65
ARL6IP4	BC015569	Nuclear, ARL6 GTP-binding IP, vesicular trafficking	0.03	0.02	0.06	0.00	0.00	0.61
NCLN	BC019091	ER, TGFbeta superfamily signal transduction	0.08	0.10	0.01	0.02	0.22*	0.62
YIPF5	BC007829	Golgi/ER, Rab GTPase	0.94	0.95	0.94	0.99	0.96	0.30
GCC1	BC014100	Golgi (TGN)	0.96	0.95	0.95	0.98	0.93	0.42
COG4	BC013347	Golgi, retention and retrieval of Golgi proteins	0.93	1.00	0.81	0.98	1.00	0.47
COPZ1	BC002849	ER-Golgi transport, non-CC vesicle coat	0.95	1.00	0.85	0.98	1.00	0.45
CTSD	BC016320	Lysosome, aspartyl protease	0.95	1.00	0.86	0.99	0.99	0.32
DPP7	BC011907	Lysosome, peptidase	0.95	1.00	0.86	1.00	1.00	0.41
CPVL	BC016838	ER, peptidase	0.94	0.99	0.84	0.99	1.00	0.28
LEPREL1	BC005029	ER/Golgi, protein metabolism	0.94	0.95	0.86	0.97	0.97	0.54
SEMA3B	BC013975	ER, growth cone guidance	0.95	0.99	0.87	0.97	0.99	0.34
CRMP1	BC007898	Semaphorin signal transduction pathway	0.91	0.99	0.71	0.94	0.99	0.31
VPS37C	BC005805	Endosome, sorting ubiquinated transmembrane proteins	0.92	1.00	0.84	0.97	1.00	0.50
*Lipid/cholesterol metabolism*								
APOL1	BC017331	HDL complex, cholesterol trafficking, secreted	0.03	0.00	0.09	0.02	0.01	0.65
APOL2	BC004395	HDL complex, cholesterol trafficking, cytoplasmic	0.97	0.99	0.91	0.94	0.98	0.30
SCARB1	NM_005505	HDL receptor	0.93	0.99	0.83	0.99	0.99	0.35
FDFT1	BC003573	ER, first step in cholesterol biosynthesis	0.92	1.00	0.83	0.99	1.00	0.47
PRKAB1	BC001823	Cell energy homeostasis, fatty acid biosynthesis	0.91	0.99	0.83	0.98	0.30**	0.50
LASS4	BC009828	ER, Ceramide synthesis	0.95	0.98	0.90	0.98	0.92	0.61
PTGES	BC018201	Prostaglandin metabolism	0.96	0.96	0.94	0.98	0.91	0.34
ZDHHC1	BC021908	Palmitoyl transferase	0.98	0.98	0.96	0.98	0.96	0.32
ZDHHC8	BC009442	Palmitoyl transferase	0.93	0.99	0.83	1.00	0.89	0.49
PHLDB1	BC013031	PtdIns(3,4,5)P(3) binding, postsynaptic membrane	0.95	0.99	0.88	0.97	1.00	0.28
PXMP4	BC001147	Peroxisomal membrane protein, lipid metabolism	0.94	0.95	0.97	0.96	0.96	0.34
ECHDC1	BC003549	Peroxisomal oxidation of fatty acids	0.06	0.04	0.14	0.00	0.01	0.51

Thirty-nine significantly regulated transcripts belonged to three distinct functional groups: calmodulin-related signaling, Golgi/ER-related transcripts and lipid/cholesterol metabolism. Group average p-values (GAPs, two-tailed *z*-test) are indicated for all 34 drug abuse cases (ALL), and for the three main groups of cases sorted according to the drugs present at the time of death: cocaine (COC+, N = 12), cannabis (THC+, N = 9) and phencyclidine (PCP+, N = 3). While factors such as brain pH, postmortem interval, gender or smoking history were all well-matched, ethnicity was the only demographic variable that differed between groups (*Table 1*). To address this issue, we examined only the six African-American male (AAM) drug abuse cases (18% of all drug abuse cases) that had been individually matched to four AAM controls. Of the 39 transcripts that were significantly regulated (ALL and COC+, THC+ and/or PCP+ columns), 37 remained similarly regulated for the AAM group. There were only two transcripts, NCLN (*) and PRKAB1 (**) for which the results were different for the AAM subgroup (see text). Therefore, as a factor in matching, ethnicity had little effect on the identification of significantly regulated transcripts. Controls with cardiovascular disease (CVDC) and elevated body mass index (*Table S1*) did not demonstrate changes in gene expression which were similar to those of the drug abuse cases. Table S2 contains the full list of transcripts. Gene symbols were annotated using GenBank Accession numbers and EntrezGene.

#### Cardiovascular disease

Each of six overweight-obese controls with cardiovascular disease (CTR 5, 7, 10, 11, 14, 26) were compared to the best-matched three cases from a group of six normal-overweight control cases with no history of cardiovascular disease (CTR 12, 23, 27, 30, 31, 32), as indicated in Table S1. Microarrays were processed at the same time for these and the drug abuse cases. GAP scores are included for transcripts selected for the drug abuse cases (*Table 3*, *Table S2*).

### Annotation and nomenclature

Data were annotated using Source BatchSearch (http://genome-www5.stanford.edu/cgi-bin/source//sourceBatchSearch), and EntrezGene cross-database search (http://www.ncbi.nlm.nih.gov/gquery/gquery.fcgi). Gene names and symbols follow the HGNC nomenclature employed by EntrezGene.

### Quantitative real-time PCR (QPCR)

RNA from a separate dissection of aPFC cortical tissue from all drug abuse and control cases was DNase-treated (Qiagen Inc., Valencia, CA) and evaluated using the Bioanalyzer. As previously described [14], RNA was reverse transcribed using AMV reverse transcriptase (Roche Molecular Biologicals, Indianapolis, IN). A commercial 18S RNA primer kit (Ambion, Austin, TX) was used to quantify the amount of cDNA examined. Primers were designed using MacVector software (Accelrys, San Diego, CA), and synthesized by Gene Probe Technology (Gaithersburg, MD): APOL2(F): aac cgc cac gat aaa gac cag; APOL2(B): cac cca caa act cct tca tca cc; CALM2(F): gca gaa tcc cac aga agc aga g, CALM2(B): gcc att gcc atc ctt atc aaa; SEMA3B(F): aga ctt tca gcc tgg agc gaa c, SEMA3B(B): gca aat ggg tgc ggt tgt ag.

QPCR was performed using the DyNAmo® HotStart SYBR Green QPCR kit and the Opticon DNA Engine (MJ Research, Waltham, MA), using 15 min pre-incubation and 40 cycles of denaturation at 95°C, annealing at 60°C, and extension at 72°C (each for 30 seconds). Only data within the linear range of the assay were used. A single product of the expected size was confirmed by melting curve analysis and gel electrophoresis. Individual samples were selected based on *p*-values. The ratio between individual drug abuse cases and their four best-matched controls were calculated for both QPCR experiments and for microarray data. For the latter data, fold changes were calculated from data scaled to the same average hybridization intensity. The data represents an average of three independent experiments. The limiting factor in the number of transcripts and individuals examined was the amount of control cDNA available.

## Results

### Groups defined by hierarchical clustering of global transcriptional profiles

Hierarchical clustering is a natural first step in microarray analysis, where it enables a global view of complex microarray data. This information on the overall similarities and differences in gene expression reveals intrinsic groupings with similarities in transcriptional profiles. For human postmortem cases, which rely heavily on anecdotal information from next-of-kin, the medical and drug abuse history may not be fully known or disclosed, which may significantly influence the transcriptional profile, and hierarchical clustering thus provides an additional quality control prior to a full comparative analysis.

In the present study, hierarchical clustering of microarray z-ratio data identified three main transcriptional groups (*Fig. 1*). Eight drug abuse (DA) cases (DA35–DA42, Group 3) were discriminated at the first tier, suggesting that these cases were distinctly different from the remaining 34 cases, which separated into Group I (DA1–DA18), and Group II (DA19–DA34).

Juxtaposing drug abuse history and toxicology for each case with the clustering dendrogram did not clearly distinguish groups by differences in lifetime drug abuse history or by the nature of drugs present at death (*Fig. 1*). A clear distinctions between “cocaine”, “cannabis” and “phencyclidine” cases may not even have been possible by global transcriptional profiling, as a significant polysubstance abuse, mostly of cocaine, cannabis and/or phencyclidine, was uncovered in the drug abuse history and toxicological examination. Subgroups within Groups I–III did, however, appear somewhat related to drug abuse history or toxicology, as illustrated by a clustering of six cases with a cocaine/crack-cocaine abuse history (DA2–7), and of two cases with similar blood alcohol levels (DA12–13).

Overall, a past or present abuse of cocaine was indicated by the drug abuse history and/or general and hair toxicology for a large proportion (32/42, 76%) of the cases (*Fig. 1* *,* *Table 2*, *Table S1*). Significant blood alcohol levels (≥0.05 g/dL (g%)) were more prominent in Group I (9/18, 50%) than in Group II cases (3/16, 19%). Conversely, opioids were not found in Group I cases, but were present in 5/16 (31%) Group II cases. Furthermore, Group II contained 5/9 (56%) of the phencyclidine cases, while Group I and Group II each had two cases (22%).

Group III cases appeared different from Group I and Group II cases, in that they represented cases with no or only residual cocaine, cannabis or phencyclidine and metabolites, presented with significant other medical circumstances, such as anaphylactic shock, major depression, suicide completion, and organic disease related to chronic alcoholism. Five cases (DA36, DA38–41) were primarily described as alcohol dependent. The three remaining cases (DA35, DA37, DA42) were characterized as cannabis users by history, but general toxicology and hair testing found additional substances, while neuropathological examination revealed cerebral ischemic changes and/or brain edema.

### Distribution of individual changes in groups defined by global transcriptional profiles

**Figure 2:**
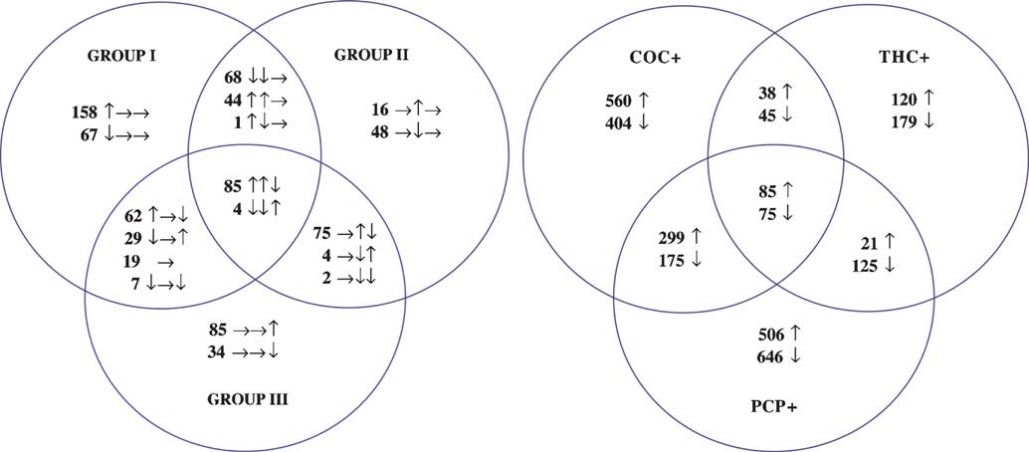
Venn Diagrams illustrating the distribution of significantly altered transcripts in groups defined by global expression profiles (A) or by drugs of abuse (B). *A.* Eighty-nine transcripts were regulated in all three groups defined by global expression profiles. All of these were regulated in the same direction (increased or decreased) for Group I (DA1–18) and Group II (DA19–34), and in the opposite direction for Group III (DA35–42) cases. In all, 201/202 transcripts shared between Group I and Group II were regulated in the same direction. For Group III, 91/115 transcripts shared with Group I and 79/81 transcripts shared with Group II were regulated in the opposite direction. These data highlight the similarities of Group I and Group II, and the marked differences in Group III cases. Each cluster of three arrows indicates the direction of change in Groups I, II and III, respectively. Increased expression is indicated by ↑, a decrease by ↓, while → indicates no significant change. *B.* Cases with a drug abuse history and positive cocaine, phencyclidine or cannabinoid toxicology in blood, brain or urine were grouped into COC+, PCP+ and THC+ groups, respectively. While there were significant transcriptional differences between these groups, a total of 160 transcripts (≈2% of all transcripts) were shared for the three groups. Note that a much smaller number of transcripts were identified for the THC+ group (264 increased, 424 decreased) than for the COC+ (982 increased, 699 decreased) and PCP+ (911 increased, 1021 decreased) groups. Increased expression is indicated by ↑, decreased expression by ↓.

The distinct differences in Group III compared to Groups I and II described above were further confirmed by examination of changes in individual transcripts for these groups (*Fig. 2A*). Group average *p*-value (GAP) criteria, based on a two-tailed *z*-test comparing z-scores for each drug abuse case to the four individually best-matched controls, was employed to identify individual transcripts for which expression was either increased or decreased in at least one of the groups identified by hierarchical clustering above.

Of 808 individual transcripts identified (*Fig. 2A*), 89 were significantly changed in all three groups. All were regulated in the same direction for Groups I–II, but in the opposite direction for Group III. Expression of 85 transcripts, including APOL2, GCC1, PTGES, were increased for Groups I–II and decreased for Group III. TYMS, C5orf3, PTRF and KLF5 were decreased in Groups I–II and increased in Group III.

An additional 113 transcripts were significantly changed in Groups I–II only; 44 transcripts, including FIBP, VTN, STOM, ICAM1, SEMA3B, RARG, RARA, were increased, and 68 transcripts, including APOL1, CALM1, CALM2, CAMK2B, GNB2L1, HINT1, RAB9A, CHEK1, PPP2CA and NFKBIA, were decreased. Only one transcript, SP100, was increased in Group I and decreased in Group II. Consequently, 201 of the 202 transcripts significantly changed in Groups I and II were changed in the same direction.

*In conclusion*, compared to Groups I–II, the eight Group III cases represented different medical and lifetime drug use histories and toxicological profiles (*Fig. 1*). While the exact nature of these differences could not be causally linked to specific ante or postmortem factors, alcohol dependence appeared to be a primary diagnosis for 5/8 Group III cases, which along with no or low concentrations of cocaine, cannabis or phencyclidine and significant other medical problems may have led to opposite regulation in Group III of the 89 transcripts shared among all three groups, of 91/115 transcripts changed in both Group I and Group III, and of 79/81 transcripts changed in both Groups II and III.

As Group III cases represented a small minority with distinctly different features, we focused the study on the 34 Group I and II cases.

### Transcriptional regulation in groups defined by toxicology and drug use history

To identify transcripts that were similarly regulated by cocaine, cannabis and phencyclidine, we again employed GAP scores to examine 24 Group I–II cases with a confirmed drug abuse history and toxicological evidence of cocaine, cannabis or phencyclidine (COC+, THC+ and PCP+, respectively) present at death. This identified 160 transcripts shared between the COC+, THC+ and PCP+ groups (*Fig. 2B*). Additionally, 474 transcripts were shared by the COC+ and PCP+ groups, 83 transcripts by the COC+ and THC+ groups, and 146 transcripts by the PCP+ and THC+ groups. A total of 964, 299 and 1152 transcripts were exclusively changed in the COC+, THC+ and PCP+ groups, respectively. The lower total number of transcripts identified for cannabis cases was noteworthy.

*In conclusion*, while there were significant differences in transcriptional regulation between cocaine, cannabis or phencyclidine abuse cases, a similar transcriptional regulation extended across drug classes and cases for 160 transcripts.

### Selection and functional annotation of transcripts shared across specific drugs of abuse

To select transcripts that would reflect effects of drug use irrespective of which specific drug(s) were used, only transcripts that by GAP criteria were significantly changed in the majority (30/34) of Group I–II drug abuse cases and in two of the three drug-defined groups (COC+, THC+, and PCP+) were included. After exclusion of cDNA clones removed from GenBank, the list contained 139 transcripts, which were sorted according to function and location (*Table S2*). Three functional groups, comprising 39 transcripts (*Table 3*), are discussed below.

### Calmodulin-related signaling

A general decrease was observed for calcium/calmodulin-regulated transcripts (CALM1, CALM2, CAMK2B, S100A16) and a transcript encoding a phosphatase putatively involved in cAMP biosynthesis and adenylate cyclase (AC) activity (THTPA).

### Golgi/ER-related transcripts

Transcripts involved in various aspects of Golgi/ER function (YIPF5, GCC1, COG4, COPZ1, VPS37C, LEPREL1, CPVL, SEMA3B) were increased. Also increased were transcripts encoding adaptins and an adaptin kinase (AP1M2, AP2A1, AP4B1, AAK1) associated with clathrin-coated vesicle endocytosis at the plasma membrane and in transport between the Golgi apparatus and the endosomal system. While transcripts encoding lysosomal enzymes (CTSD, DPP7) were increased, transcripts encoding proteins involved in synaptic vesicular trafficking (RIMBP2, ARL6IP4), transport from the late endosome to the lysosome (ASGR2, RAB9A), and a marker of cholesterol- and sphingolipid-rich lipid raft membrane microdomains involved in clathrin-independent endocytosis (FLOT1) were all decreased.

### Lipid/cholesterol metabolism

Most transcripts regulating cholesterol biosynthesis (FDFT1, PRKAB1) and trafficking (SCARB1, APOL2) were increased, but transcription of the secreted apolipoprotein APOL1 and a peroxisomal protein (ECHDC1) involved in beta-oxidation of cholesterol were decreased. Expression of the LASS4, PTGES, ZDHHC1 and ZDHHC8 transcripts involved in lipid biosynthesis, metabolism and modification were all increased.

### Quantitative Polymerase Chain Reaction (QPCR)

**Figure 3:**
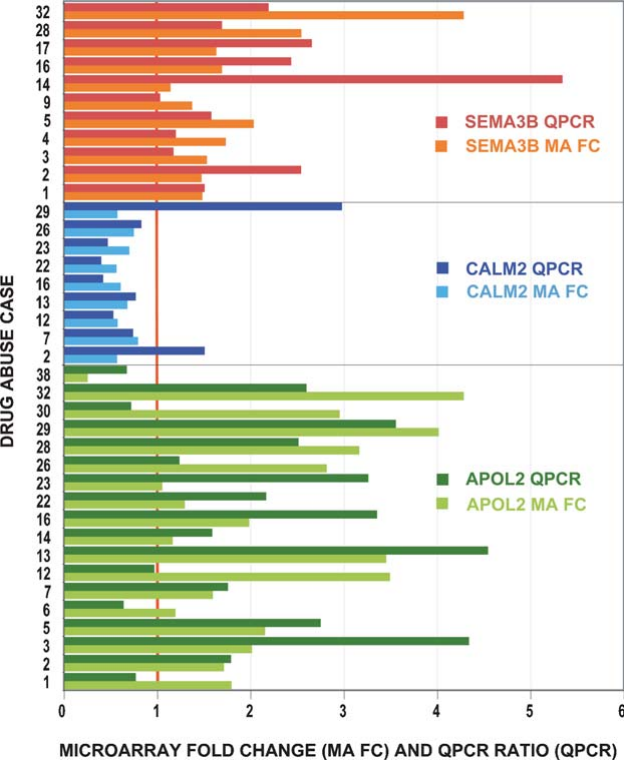
Validation of microarray data by quantitative PCR (QPCR). Three representatives of consistently changed functional groups (Table 3) were examined by QPCR: calmodulin 2 (CALM2, blue bars), apolipoprotein 2 (APOL2, green bars), and semaphorin 3B (SEMA3B, orange bars). Numbers on the x-axis represents either the microarray-derived fold change (FC, lighter blue, green or orange bars) for each drug abuse case compared to the four best-matched control cases or the QPCR ratio (QPCR, darker blue, green or orange bars), while numbers on the y-axis represent the drug abuse case examined. QPCR validated 32/38 (84%) of the microarray data, which was performed using separate sets of brain dissections and RNA extractions from each drug abuse case and the four individual best-matched controls.

Transcripts representing three consistently changed functional classes were examined using QPCR (Fig. 3). These experiments were performed in triplicate for each drug abuse case and their individual four best-matched controls, using different aPFC dissections and RNA extractions than those used for microarray experiments. Microarray data were expressed as fold changes between individual drug abuse and control cases to facilitate a more direct comparison to similar ratio data from QPCR analysis of the same drug abuse and control cases.

Calmodulin 2 (CALM2) expression (Fig. 3, blue bars) was decreased in two microarray CALM2 clones. QPCR validated decreased CALM2 expression in 7 of 9 drug abuse cases.

Apolipoprotein L, 2 (APOL2) expression (Fig. 3, green bars) was increased by microarray analysis. QPCR validated the increase in 13 of 17 cases. To examine if decreases detected by microarray analysis were demonstrable by QPCR, one Group III case, DA38, was employed. The decreased expression for this case was validated.

Semaphorin 3B (SEMA3B) expression (Fig. 3, orange bars) was increased by microarray analysis, which was validated in 11 of 11 cases examined by QPCR.

While some variability was expected, given that separate dissections were employed for microarray analysis and QPCR experiments, the general pattern and degree of difference detected by the two techniques were very similar, and results agreed in 32/38 cases (84%).

### Specificity of these findings with respect to ethnicity

While there was similar matching of the drug abuse and control groups for five (*brain pH, PMI, age, gender and smoking history*) potential confounding factors (*Table 1*), a sixth matching factor (*ethnicity*) differed, and a separate analysis was performed to address this.

As 36 of the drug abuse cases were African-American (86%) and most were male (33/42, 79%), we examined the average statistical significance of differences (Group average *p*-value, GAP, two-tailed *z*-test) between drug abuse and control cases for the six drug abuse cases for which both drug abuse and all four control cases were male and of African-American origin (AAM). Even though this analysis employed only ∼1/6 of the 34 drug abuse cases analyzed (*Table 3*), 37 of 39 transcripts analyzed remained significant at a similar level. For NCLN, the average *p*-value was 0.22, due to one outlier, but the median was 0.00 for the six AAM cases, indicating that this transcript was similarly changed for most of the cases. For PRKAB1, the average *p*-value decreased from *p* = 0.91 for all 34 cases to *p* = 0.30 for the six AAM drug abuse cases (median *p* = 0.03). Thus, 38 of 39 transcripts were similarly changed when comparing all cases to the six AAM drug abuse cases. Therefore, the ethnicity of the cases did not exert a major influence on the identification of significantly changed transcripts across drug abuse cases.

### Specificity of these findings to drug abuse

In order to determine whether these changes were specific to drug abuse or might reflect a common physiological response to cardiovascular disease, as common complication in drug abuse cases, or oxidative stress secondary to drug abuse, we examined control cases with or without cardiovascular disease. Six overweight-obese control cases with cardiovascular disease and six normal-overweight control cases with no history of cardiovascular disease were compared. GAP scores did not reach significance for any of transcripts identified to be increased or deceased in drug abuse cases (*Table 3*, *Table S2*).

## Discussion

The prefrontal cortex is centrally involved in the neurobiological circuitry that promotes drug use and compulsive drug-seeking behavior, and appears to be similarly involved for various drugs of abuse [3], [4]. We therefore hypothesized that multiple types of drug abuse would share common transcriptional changes, due to similar changes in cellular function. We tested this using microarray analysis of postmortem aPFC from 42 cases with varied drug abuse histories as determined by postmortem case history reviews, next-of-kin interviews, and toxicological examination.

### Global transcriptional changes

Hierarchical clustering of the global transcriptional profiles and examination of changes in individual transcripts demonstrated consistent changes in gene expression in a majority of cases (N = 34). A subgroup of eight drug abuse cases displayed markedly different, and mostly opposite, gene expression changes which appeared to reflect distinct differences in lifetime drug abuse histories, toxicology and other medical conditions.

### Functional annotation

Based on the cohort of 34 drug abuse cases, we identified and assigned function and/or location to 139 transcripts (*Table S2*). None of these transcripts were significantly altered by the combined cellular stress of cardiovascular disease and elevated body mass index (*Table 3*, *Table S2*). Three distinct functional categories (*Table 3*) were selected for further discussion in the context of their potential impact on aPFC function.

### Calmodulin signaling

Transcripts encoding calcium/calmodulin-related signaling transcripts were decreased, which was validated for CALM2 by QPCR (*Fig. 3*). Studies in human postmortem brain have previously demonstrated a general decrease in calcium/cAMP-related signaling transcripts in frontal and motor cortex of human alcoholics [13], a decrease in adenylate cyclase (AC) type I (AC-I) protein levels and activity in temporal and frontal cortex in alcoholics [22]–[23] and in AC-I mRNA and protein in temporal cortex from heroin addicts [24]. While AC-I protein levels were unchanged in prefrontal cortex from opiate addicts, there were significant decreases in the downstream MAPK signaling cascade [25]. Furthermore, methamphetamine administration decreased calcium/calmodulin-dependent protein kinase II (CAMKII) activity and phosphorylated (but not total) CAMKIIα in rat frontal and parietal cortex [26].

Since calmodulin has been hypothesized to be a central integrator of synaptic plasticity [27] and modulates the activity and calcium-sensitivity of key signaling molecules, such as AC, CAMKII and IV (CAMKIV), and the Ras-MAPK signaling pathway, this effect could significantly impact a number of downstream signaling pathways. One such target is CAMKII, which is critical for the establishment of synaptic plasticity and memory and in stabilizing dendritic architecture [28], with a specific role for CAMKIIB in targeting the alpha CAMKII isoform to dendritic spines and the cell cortex [29]. The significant decrease in CALM1, CALM2 and CAMKIIB transcripts suggest that altered regulation of neuroplasticity and dendritic architecture by calmodulin-regulated pathways could be a common theme in human abuse of cocaine, cannabis and/or phencyclidine.

### Lipid and cholesterol metabolism: Apolipoprotein L family

Increased transcription was demonstrated for transcripts related to lipid and cholesterol metabolism, except for two transcripts encoding proteins that reduce available cholesterol in the cell by excretion or metabolism. Expression of transcripts encoding the first specific step in the cholesterol biosynthetic pathway (FDFT1), the high-density lipoprotein (HDL) receptor (SCARB1) and an inhibitor of cholesterol biosynthesis (PRKAB1) were increased. Of particular note were increases in the apoliprotein L (APOL) transcripts, for which the increased expression of APOL2 was validated by QPCR (*Fig. 3*). APOL family proteins are HDLs, which play a central role in cholesterol transport and homeostasis. The six known APOL genes are exclusively present in primates, with the APOL1-4 genes forming a tight cluster on human chromosome 22q13.1 [30]. The APOL2 transcript is most highly expressed in brain, while the APOL1 transcript differs from other APOL transcripts by an additional exon that produces a secreted APOL protein [30]. These differences may also underlie the opposite changes in the APOL1 and APOL2 transcripts in the drug abuse cases. APOL4 was significantly increased only in the cocaine cases.

Cholesterol is indispensable for neuronal functioning, synaptic plasticity and CNS myelination [31]–[32]. Functionally, this may also relate to the decreased frontal white matter volume observed in drug abusers [33]. Normal white matter maturation appears to be arrested in the frontal and temporal lobes in cocaine abusers [34]. Microarray studies have demonstrated changes in myelin transcripts in the nucleus accumbens and prefrontal cortex of cocaine abusers [14], [16]. This is reminiscent of the situation in the prefrontal cortex in schizophrenia, where expression of APOL1, 2 and 4 transcripts were increased [35], and myelin transcripts were decreased [9]. Importantly, changes in these primate-specific lipid metabolism genes underscore inherent differences between non-primate models and human drug abuse at a cellular level, and highlight the importance of studies in human postmortem brain.

### Intracellular trafficking and the ER/Golgi compartments

A number of transcripts encoding proteins regulating intracellular trafficking of secretory pathways were changed, with a predominant increase in transcription of ER/Golgi-related transcripts. Increased transcription of the ER-resident protein semaphorin 3B (SEMA3B) was validated by QPCR (*Fig. 3*). This increase mirrors the significant cocaine-induced increases of SEMA3B mRNA in the hippocampus and nucleus accumbens of cocaine-sensitized or chronically cocaine-treated animals [36]. Secreted semaphorins play critical roles in growth cone guidance, axonal positioning and in modulating apical cortical dendrites [37]–[39], dendritic branching and spine maturation [39] and may modulate synaptic transmission in the adult brain [40]. Transcription of clathrin adaptors and an adaptor-associated kinase functioning in clathrin-coated carrier vesicles between the trans-Golgi network and the endosomal system, and endocytic vesicles at the plasma membrane were also consistently increased. Transcripts for which expression was decreased were mainly associated with synaptic vesicular trafficking, clathrin-independent endocytosis or transport from the late endosome to the lysosome.

Neurons have a unique organization of their secretory pathway, with membrane and lipid processing in somatic Golgi, and additional ER-to-Golgi transport in dendrites [41], which enables dendrites with Golgi to be longer and more complex [42]. In that context, the shared increases in transcription of genes relating to ER and Golgi synthesis and processing, and decreased transcription of genes invested in other transport functions is interesting, and suggests that changes in aPFC dendritic function and plasticity is shared among drug abuse cases.

### Implications

It has been established that drugs of abuse such as cocaine, amphetamine and morphine impart an experience-dependent structural plasticity that alters dendritic length and complexity in brain areas associated with persistent drug-induced changes [43]–[45], changes which may additionally limit later experience-dependent structural plasticity [46].

The aPFC is distinguished from other cortical regions by its highly complex synaptic connectivity and dendritic arborization [5]–[6]. Therefore, the specific changes in transcripts involved in calmodulin-regulated signaling, cholesterol metabolism and ER/Golgi function may be correlates of drug-induced changes in neuronal function and synaptic plasticity that are shared by cocaine, cannabis and phencyclidine abuse.

## Supporting Information

Table S1 Essential demographic data for the drug abuse. Each drug abuse case was matched to four controls as indicated in the control group column by brain pH, postmortem interval (PMI, hours), age (years), ethnicity (A - Asian, AA - African-American, CAUC - Caucasian, HISP - Hispanic), gender (F - female, M - male) and smoking history (YES or NO). Manner of death was accidental (A), natural (N), a homicide (H) or suicide (S). Control cases with CVD (CTR12, 23, 27, 30–32) were compared to control cases without CVD (CTR5, 7, 10, 11, 14, 26), as indicated in italics in the control group column, to examine the effect of cardiovascular disease on gene expression. Other abbreviations: ASCVD - atherosclerotic CVD, CVD - cardiovascular disease, GSW - Gunshot wound, HCVD - hypertensive CVD, MGSW - multiple GSWsst of transcripts. Gene symbols were annotated using GenBank Accession numbers and EntrezGene.
(0.04 MB DOC)

Table S2 Full list of significantly regulated transcripts in postmortem aPFC from cocaine, phencyclidine and/or cannabis abusers. One-hundred and thirty-nine transcripts were significantly regulated by group average p-value (GAP) criteria. Overweight/obese controls with cardiovascular disease (CVDC) compared to normal/overweight controls (Table S1) illustrated that drug-induced cellular stress was not an underlying cause in the common transcriptional patterns in the drug abuse cases. Gene symbols were annotated using GenBank Accession numbers and EntrezGene.
(0.06 MB DOC)
