# Supplementary material for: Transcriptional Changes Common to Human Cocaine, Cannabis and Phencyclidine Abuse
Source: PLoS One. 2006 Dec 27;1(1):e114. doi: 10.1371/journal.pone.0000114 (PMC1762434; doi:10.1371/journal.pone.0000114)
Supplement: Table S1 — Essential demographic data for the drug abuse. Each drug abuse case was matched to four controls as indicated in the control group column by brain pH, postmortem interval (PMI, hours), age (years), ethnicity (A - Asian, AA - African-American, CAUC - Caucasian, HISP - Hispanic), gender (F - female, M - male) and smoking history (YES or NO). Manner of death was accidental (A), natural (N), a homicide (H) or suicide (S). Control cases with CVD (CTR12, 23, 27, 30–32) were compared to control cases without CVD (CTR5, 7, 10, 11, 14, 26), as indicated in italics in the control group column, to examine the effect of cardiovascular disease on gene expression. Other abbreviations: ASCVD - atherosclerotic CVD, CVD - cardiovascular disease, GSW - Gunshot wound, HCVD - hypertensive CVD, MGSW - multiple GSWsst of transcripts. Gene symbols were annotated using GenBank Accession numbers and EntrezGene. (0.04 MB DOC) [file pone.0000114.s001.doc]

# **Case Gender Age pH PMI Ethnicity Manner and Cause of Death Nicotine use Control group**

**DA1** F 17 6.95 15.5 AA H GSW YES CTR 5, 7, 21, 29

**DA2** M 19 6.91 13.5 AA H GSW YES CTR 5, 7, 21, 23

**DA3** M 30 6.58 28.0 AA H GSW NO CTR 1, 10, 25, 26

**DA4** M 50 6.93 14.0 AA N HCVD NO CTR 8, 12, 26, 29

**DA5** M 40 6.55 29.0 AA H Stab wound NO CTR 1, 10, 25, 26

**DA6** M 50 6.59 12.5 AA N Acute intoxication YES CTR 7, 12, 15, 23

**DA7** M 28 6.70 34.0 AA H GSW NO CTR 8, 10, 15, 23

**DA8** M 26 6.98 23.5 AA H GSW YES CTR 5, 8, 21, 23

**DA9** M 47 6.67 21.5 HISP N Acute MI NO CTR 11, 24, 25, 26

**DA10** M 23 6.87 34.0 AA H MGSW YES CTR 5, 23, 28, 29

**DA11** M 18 6.80 21.5 AA H MGSW NO CTR 5, 11, 21, 28

**DA12** M 31 6.91 22.0 AA H Stab wound NO CTR 8, 21, 23, 29

**DA13** M 42 6.93 33.5 AA H Stab wound YES CTR 8, 18, 21, 23

**DA14** M 21 6.72 14.5 AA H GSW YES CTR 7, 12, 13, 23

**DA15** F 47 6.63 23.0 CAUC N Upper GI hemorrhage YES CTR 7, 12, 15, 23

**DA16** M 29 6.70 14.0 AA H Stab wound YES CTR 7, 13, 23, 25

**DA17** M 27 6.58 10.5 AA H GSW NO CTR 11, 19, 24, 28

**DA18** M 33 6.16 31.0 AA H GSW YES CTR 2, 3, 9, 17

**DA19** M 24 6.43 21.5 AA H GSW YES CTR 7, 9, 13, 15

**DA20** M 20 6.27 32.5 AA H MGW YES CTR 1, 6, 9, 13

**DA21** M 30 6.32 72.5 AA N Pneumonia YES CTR 6, 9, 16, 30

**DA22** F 42 6.47 31.0 CAUC A Acute intoxication YES CTR 7, 9, 15, 23

**DA23** M 18 6.76 11.5 AA H GSW NO CTR 5, 7, 11, 28

**DA24** F 20 6.87 15.5 AA H GSW YES CTR 5, 7, 12, 23

**DA25** M 18 7.03 43.0 AA H MGSW YES CTR 5, 8, 15, 23

**DA26** M 35 6.91 40.0 AA H Stab wound NO CTR 8, 18, 21, 26

**DA27** M 15 6.79 15.5 AA H Stab wound NO CTR 5, 11, 21, 28

**DA28** F 33 6.88 12.5 AA H GSW YES CTR 7, 21, 23, 29

**DA29** M 28 6.73 36.5 AA H GSW YES CTR 8, 15, 21, 23

**DA30** M 33 6.94 41.5 AA H GSW YES CTR 8, 18, 21, 23

**DA31** M 15 6.47 20.5 AA H GSW YES CTR 7, 9, 13, 15

**DA32** M 28 6.98 24.0 AA H MGSW NO CTR 5, 21, 23, 29

**DA33** F 47 6.92 66.0 AA A Acute intoxication YES CTR 9, 18, 21, 27

**DA34** F 51 6.75 20.0 HISP A Acute intoxication NO CTR 24, 25, 26, 29

**DA35** M 30 6.78 11.5 AA H GSW NO CTR 11, 19, 22, 24

**DA36** M 39 6.96 19.5 AA N HCVD YES CTR 5, 12, 23, 29

**DA37** M 19 6.90 16.5 AA H GSW YES CTR 5, 7, 12, 23

**DA38** M 45 6.82 18.5 AA S GSW YES CTR 5, 7, 12, 23

**DA39** M 40 6.10 22.0 AA H Stab wound YES CTR 2, 4, 17, 20

**DA40** M 35 6.79 35.0 CAUC N Alcoholism YES CTR 8, 15, 23, 25

**DA41** M 45 6.79 13.0 AA N Anaphylactic shock NO CTR 7, 11, 12, 29

**DA42** M 17 6.93 39.0 AA H GSW NO CTR 5, 8, 14, 21

**CTR1** M 20 6.46 25.5 AA H GSW NO -

**CTR2** M 58 6.07 15.0 AA N ASCVD YES -

**CTR3** F 57 6.00 45.5 AA N Pneumonia YES -

**CTR4** M 57 6.27 24.0 CAUC N Occlusive coronary atherosclerosis NO -

**CTR5** M 19 7.01 21.0 HISP H Stab wounds NO *-*

**CTR6** M 58 6.27 37.0 A H Stab wound YES -

**CTR7** M 18 6.62 11.5 AA H GSW YES *-*

**CTR8** M 29 6.88 32.5 AA H MGSW NO -

**CTR9** F 45 6.43 43.0 AA N Aortic aneurysm YES -

**CTR10** M 36 6.55 36.5 AA A Chest trauma NO *-*

**CTR11** M 27 6.59 19.5 CAUC A Asphyxia NO *-*

**CTR12** M 60 6.69 13.0 AA N ASCVD YES *CTR 7, 11, 26*

**CTR13** M 21 6.49 15.0 AA H GSW YES -

**CTR14** M 20 6.87 33.5 HISP H GSW NO -

**CTR15** M 24 6.57 36.0 CAUC N Bronchial asthma YES -

**CTR16** M 53 6.17 63.0 CAUC N ASCVD YES -

**CTR17** M 35 6.05 22.0 HISP A Blunt force injuries YES -

**CTR18** F 31 6.91 33.5 AA N Myocarditis NO -

**CTR19** M 37 6.37 13.0 AA N Subarachnoid hemorrhage NO -

**CTR20** F 39 6.10 33.0 CAUC N Viral myocarditis NO -

**CTR21** M 29 6.91 29.5 AA N Cardiomyopathy NO -

**CTR22** F 13 6.18 19.5 AA H Shotgun wound NO -

**CTR23** M 34 6.71 29.5 CAUC N Coronary atherosclerosis YES *CTR 7, 14, 26*

**CTR24** M 49 6.57 18.5 CAUC N Cardiomyopathy NO -

**CTR25** M 44 6.77 29.0 AA N Chronic bronchial asthma NO -

**CTR26** M 44 6.77 29.0 AA H Multiple stab wounds NO -

**CTR27** F 64 6.83 52.5 AA N Cardiac tamponade, ASCVD NO *CTR 5, 14, 26*

**CTR28** F 19 6.57 9.5 CAUC N Myocarditis, cardiac arrythmia NO -

**CTR29** M 46 6.83 13.0 AA N Mitral valve prolapse NO -

**CTR30** M 53 6.45 57.0 AA N HCVD YES *CTR 10, 11, 26*

**CTR31** M 58 6.87 64.0 CAUC N Aortic dissection, HCVD YES *CTR 5, 14, 26*

**CTR32** M 51 6.54 56.0 AA N ASHCVD NO *CTR 10, 11, 26*

*Table S1.* *Essential demographic data for the drug abuse (DA) and control (CTR) cases.* Each drug abuse case was matched to four controls as indicated in the control group column by brain pH, postmortem interval (PMI, hours), age (years), ethnicity (A – Asian, AA – African-American, CAUC – Caucasian, HISP – Hispanic), gender (F – female, M – male) and smoking history (YES or NO). Manner of death was accidental (A), natural (N), a homicide (H) or suicide (S). Control cases with CVD (*CTR12, 23, 27, 30-32*) were compared to control cases without CVD (*CTR5, 7, 10, 11, 14, 26*), as indicated in italics in the control group column, to examine the effect of cardiovascular disease on gene expression. *Other abbreviations*: ASCVD – atherosclerotic CVD, CVD – cardiovascular disease, GSW – Gunshot wound, HCVD – hypertensive CVD, MGSW – multiple GSWs
