# Supplementary material for: Transcriptional Changes Common to Human Cocaine, Cannabis and Phencyclidine Abuse
Source: PLoS One. 2006 Dec 27;1(1):e114. doi: 10.1371/journal.pone.0000114 (PMC1762434; doi:10.1371/journal.pone.0000114)
Supplement: Table S2 — Full list of significantly regulated transcripts in postmortem aPFC from cocaine, phencyclidine and/or cannabis abusers. One-hundred and thirty-nine transcripts were significantly regulated by group average p-value (GAP) criteria. Overweight/obese controls with cardiovascular disease (CVDC) compared to normal/overweight controls (Table S1) illustrated that drug-induced cellular stress was not an underlying cause in the common transcriptional patterns in the drug abuse cases. Gene symbols were annotated using GenBank Accession numbers and EntrezGene. (0.06 MB DOC) [file pone.0000114.s002.doc]

**EG SYMBOL ACCESSION LOCALIZATION AND FUNCTION GAPs: ALL COC+ THC+ PCP+ CVDC**

*Calmodulin-related signaling*

S100A16 BC019099 Calcium-binding protein 0.02 0.05 0.01 0.00 0.65

THTPA BC002984 Phosphatase, cAMP biosynthesis, AC activity 0.05 0.05 0.03 0.01 0.37

CAMK2B BC019070 Synaptic function and plasticity, calmodulin-modulated 0.02 0.03 0.01 0.00 0.55

CALM1 BC000454 Synaptic function and plasticity 0.06 0.01 0.17 0.04 0.45

CALM2 BC018677 Synaptic function and plasticity 0.04 0.00 0.13 0.01 0.43

CALM2 BC017385 Synaptic function and plasticity 0.05 0.03 0.13 0.01 0.47

*Golgi/ER-related transcripts*

AAK1 BC002695 Golgi/PM, CC AP2-associated kinase 0.93 1.00 0.86 0.98 0.32

AP1M2 BC005021 Golgi/PM, clathrin-coat (CC) adaptor protein 1 (AP1) 0.95 1.00 0.86 0.97 0.48

AP2A1 BC014214 Golgi/PM, CC AP2 0.96 0.95 0.96 0.97 0.36

AP4B1 BC014146 Golgi/PM, CC AP4 0.96 0.96 0.94 0.94 0.35

FLOT1 BC001146 Lipid raft/caveola-associated, endocytosis (non-CC) 0.04 0.03 0.10 0.00 0.37

RIMBP2 BC007632 Golgi, synaptic active zone, Rab3-IM-BP 0.04 0.01 0.13 0.00 0.55

RAB9A BC017265 Late endosome/lysosome, GTPase, vesicular trafficking 0.04 0.00 0.11 0.01 0.38

ASGR2 BC017251 Vesicular transport, Galact-term glycoproteins (lysosome) 0.02 0.01 0.03 0.02 0.65

ARL6IP4 BC015569 Nuclear, ARL6 GTP-binding IP, vesicular trafficking 0.03 0.02 0.06 0.00 0.61

NCLN BC019091 ER, TGFbeta superfamily signal transduction 0.08 0.10 0.01 0.02 0.62

YIPF5 BC007829 Golgi/ER, Rab GTPase 0.94 0.95 0.94 0.99 0.30

GCC1 BC014100 Golgi (TGN) 0.96 0.95 0.95 0.98 0.42

COG4 BC013347 Golgi, retention and retrieval of Golgi proteins 0.93 1.00 0.81 0.98 0.47

COPZ1 BC002849 ER-Golgi transport, non-CC vesicle coat 0.95 1.00 0.85 0.98 0.45

CTSD BC016320 Lysosome, aspartyl protease 0.95 1.00 0.86 0.99 0.32

DPP7 BC011907 Lysosome, peptidase 0.95 1.00 0.86 1.00 0.41

CPVL BC016838 ER, peptidase 0.94 0.99 0.84 0.99 0.28

LEPREL1 BC005029 ER/Golgi, protein metabolism 0.94 0.95 0.86 0.97 0.54

SEMA3B BC013975 ER, growth cone guidance 0.95 0.99 0.87 0.97 0.34

CRMP1 BC007898 Semaphorin signal transduction pathway 0.91 0.99 0.71 0.94 0.31

VPS37C BC005805 Endosome, sorting ubiquinated transmembrane proteins 0.92 1.00 0.84 0.97 0.50

*Lipid/cholesterol metabolism*

APOL1 BC017331 HDL complex, cholesterol trafficking, secreted 0.03 0.00 0.09 0.02 0.65

APOL2 BC004395 HDL complex, cholesterol trafficking, cytoplasmic 0.97 0.99 0.91 0.94 0.30

SCARB1 NM_005505 HDL receptor 0.93 0.99 0.83 0.99 0.35

FDFT1 BC003573 ER, first step in cholesterol biosynthesis 0.92 1.00 0.83 0.99 0.47

PRKAB1 BC001823 Cell energy homeostasis, fatty acid biosynthesis 0.91 0.99 0.83 0.98 0.50

LASS4 BC009828 ER, Ceramide synthesis 0.95 0.98 0.90 0.98 0.61

PTGES BC018201 Prostaglandin metabolism 0.96 0.96 0.94 0.98 0.34

ZDHHC1 BC021908 Palmitoyl transferase 0.98 0.98 0.96 0.98 0.32

ZDHHC8 BC009442 Palmitoyl transferase 0.93 0.99 0.83 1.00 0.49

PHLDB1 BC013031 PtdIns(3,4,5)P(3) binding, postsynaptic membrane 0.95 0.99 0.88 0.97 0.28

PXMP4 BC001147 Peroxisomal membrane protein, lipid metabolism 0.94 0.95 0.97 0.96 0.34

ECHDC1 BC003549 Peroxisomal oxidation of fatty acids 0.06 0.04 0.14 0.00 0.51

**EG SYMBOL ACCESSION LOCALIZATION AND FUNCTION GAPs: ALL COC+ THC+ PCP+ CVDC**

*Cytoskeletal/signal transduction*

TSLP BC016720 Cytokine, signaling through TSLPR and IL7R 0.08 0.01 0.15 0.03 0.51

ACTG1 BC001920 Actin, cytoplasmic 0.04 0.06 0.05 0.03 0.56

TUFT1 BC008301 Integrin-binding N-linked glycoprotein 0.04 0.05 0.07 0.00 0.54

PVRL4 BC010423 Ig CAM, adhesion, migration, polarization 0.96 0.99 0.87 1.00 0.33

TMEM38A BC001195 Transmembrane protein 0.96 0.98 0.96 0.97 0.31

GPIAP1 BC001731 GPI-anchored membrane protein, Caprin-1 0.98 1.00 0.93 0.99 0.37

GIT2 BC014223 GAP, PI GTPase, cytoskeletal signaling integrator 0.96 0.92 0.96 0.96 0.39

OSGEPL1 BC011904 May regulate endomucin (focal adhesion kinase) 0.96 0.97 0.95 0.90 0.35

BLNK BC018906 Intracellular signaling, protein tyrosine kinase adaptor 0.93 0.99 0.80 0.98 0.53

KIF2C BC008764 Microtubule depolymerization, mitotic chromatin 0.93 0.98 0.81 0.98 0.36

MASTL BC009107 Microtubule-associated serine/threonine kinase 0.94 0.97 0.86 0.99 0.23

ODF2 BC010629 Centrosomal, microtubule-binding 0.95 1.00 0.87 0.98 0.27

FIBP BC014388 Mitochondrial/nuclear membrane, binds aFGF 0.95 0.99 0.87 0.99 0.33

*Ubiquitin/proteasome*

PSMA7 BC004427 Proteasome, ubiquitin-dependent protein catabolism 0.06 0.02 0.05 0.01 0.55

PSMB8 BC001114 Proteasome, ubiquitin-dependent protein catabolism 0.10 0.09 0.03 0.03 0.58

FLJ36180 BC015684 Ubiquitin ligase complex 0.03 0.01 0.09 0.00 0.59

USP3 BC018113 De-ubiquination 0.94 1.00 0.80 0.96 0.38

ADCK2 BC014107 Ubiquinone biosynthesis 0.97 0.96 0.97 0.95 0.33

TRIM65 BC013181 Protein ubiquination 0.97 0.97 0.94 0.96 0.35

*Nuclear receptors*

NR3C1 BC015610 Nuclear receptor (NR), glucocorticoid receptor 0.94 0.99 0.86 1.00 0.35

RARG BC019098 Nuclear receptor (NR), retinoic acid receptor, gamma 0.96 0.99 0.98 0.84 0.50

ZNF653 BC016816 Transcriptional repression of nuclear receptors 0.05 0.03 0.13 0.02 0.42

THRAP5 BC017282 Thyroid hormone NR-associated, transcript activation 0.04 0.02 0.07 0.00 0.63

*Transcriptional regulation*

CTCF BC014267 Transcription activator/repressor, imprinted expression 0.96 0.99 0.94 1.00 0.50

YBX1 BC010430 Transcription and RNA processing, CTCF-modulated 0.93 1.00 0.77 0.96 0.26

ZNF43 BC006528 Transcriptional repressor, HTF6 0.95 0.99 0.86 0.98 0.45

SSX2 BC007343 Transcriptional repression, Rab3IP interaction 0.96 0.99 0.94 0.83 0.30

SND1 BC017180 Activator of STAT/NfKB signal transduction 0.93 1.00 0.99 0.80 0.45

LBH BC012373 Transcription factor, organogenesis 0.95 0.99 0.87 0.96 0.35

GTF2F1 BC013007 General transcription factor 0.98 0.97 0.97 0.97 0.31

ZNF259 BC012162 Cytoplasmic, mitogens induce translocation to nucleus 0.92 0.99 0.87 0.95 0.44

STK40 BC008344 Negative regulation of NFkB/p53-mediated transcription 0.05 0.04 0.14 0.02 0.65

TSC22D1 BC000456 TGF-beta and PPARgamma-induced early TF 0.08 0.07 0.13 0.02 0.49

ZNF629 AB002324 DNA binding 0.04 0.03 0.10 0.00 0.57

BTBD2 BC000564 Cytoplasmic bodies, binds topoisomerase I 0.06 0.11 0.03 0.04 0.44

ETF1 BC014269 Cytoplasmic, translational termination 0.05 0.12 0.01 0.00 0.42

PTRF BC008849 Cytoplasmic, release of rRNA/PolI after translation 0.06 0.03 0.05 0.37 0.68

TYMS BC002567 Maintains dTMP pool for DNA replication and repair 0.07 0.02 0.03 0.34 0.74

**EG SYMBOL ACCESSION LOCALIZATION AND FUNCTION GAPs: ALL COC+ THC+ PCP+ CVDC**

*Transcriptional regulation, continued*

RPA2 BC021257 Required for DNA recombination, repair and replication 0.04 0.02 0.11 0.01 0.46

MCM2 BC017490 Pre-replication complex 0.97 0.95 0.97 0.96 0.32

HIST3H2A BC001193 Histone 2A family 0.96 1.00 0.84 0.99 0.34

DHPS BC014016 Hypusine biosynthesis, hypusine exclusively in EIF5A 0.95 0.99 0.87 0.95 0.30

CHAF1B BC021218 Chromatin assembly and repair 0.97 0.97 0.95 0.97 0.74

FTSJ2 BC017106 Nucleolar, rRNA processing, DNA repair and cell cycle 0.98 0.99 0.96 0.97 0.32

VARSL BC009355 Transcription, DNA repair 0.94 0.99 0.84 0.99 0.33

VARSL BC008844 Transcription, DNA repair 0.93 1.00 0.75 0.99 0.31

CPSF4 BC003101 Processing of 3’ pre-mRNA 0.94 1.00 0.83 0.96 0.33

TAF11 BC021972 RNA polymerase II transcription factor activity 0.94 0.93 0.97 0.98 0.66

LSM2 BC009192 Spliceosome 0.95 0.97 0.91 0.94 0.43

PRPF4 BC001588 Spliceosome 0.95 0.98 0.93 0.98 0.32

KLHDC3 BC001789 Meiotic recombination 0.94 0.94 0.90 0.97 0.33

SCYL1 BC009967 Centrosome, Serine/threonine kinase 0.95 1.00 0.84 0.99 0.34

NIP7 BC015941 Nucleolar protein impt for 60S ribosomal biosynth 0.95 0.95 0.95 0.88 0.30

RPS10 BC001955 Ribosomal protein, 40S subunit, S10E family 0.93 1.00 0.84 0.98 0.33

RPS6KB2 BC006106 Ribosomal S6 serine/threonine kinase (p90 RSK) 0.92 0.99 0.84 0.96 0.47

*Apoptosis-related*

THAP2 BC008358 Apoptosis 0.03 0.00 0.10 0.01 0.76

FANCC BC015748 Anti-apoptotic, promoting DNA repair 0.08 0.01 0.05 0.35 0.67

DIDO1 BC014489 Apoptosis 0.05 0.00 0.05 0.09 0.60

TNFRSF6B BC017065 Anti-apoptotic, helicase activity 0.93 0.99 0.82 0.98 0.43

KIAA1967 BC018269 Apoptosis, calcium ion-binding 0.93 1.00 0.84 0.96 0.51

*Mitochondrial/energy metabolism, transporters*

GRHPR BC000605 Mitochondria, metabolizing glyoxylate to glycolate 0.03 0.03 0.03 0.03 0.46

TOMM20 BC009886 Mitochondrial outer membrane translocase complex 0.05 0.03 0.10 0.00 0.51

MRM1 BC009416 Mitochondrial RNA methyltransferase activity 0.91 1.00 0.71 0.97 0.25

PC BC011617 Mitochondrial lipo-/gluconeogenesis, glutamate synthesis 0.97 1.00 0.99 0.88 0.49

PFKM BC013298 Mitochondrial, regulation of glycolysis 0.92 0.99 0.82 0.97 0.21

MRPL4 BC009858 Mitochondrial ribosomal protein 0.95 1.00 0.86 0.92 0.46

MRPL34 BC000071 Mitochondrial ribosomal protein 0.94 1.00 0.86 0.98 0.41

SLC25A3 BC011641 Mitochondrial phosphate-carrier 0.93 1.00 0.85 0.97 0.46

SLC19A1 BC003068 Folate transporter 0.95 0.99 0.87 0.97 0.35

*Unknown function*

C11orf68 BC010512 Unknown 0.94 0.99 0.81 0.98 0.25

CCDC47 BC013600 Unknown, GK001 0.95 0.99 0.87 0.96 0.33

DKFZP761I2123 BC008049 Unknown 0.95 0.97 0.97 0.97 0.31

DUSP23 BC001140 Unknown, phosphatase 0.06 0.03 0.13 0.00 0.42

C2orf15 BC021264 Unknown 0.93 0.92 0.96 0.98 0.36

C3orf29 BC001674 Unknown 0.98 0.99 0.97 0.94 0.31

C3orf34 BC007827 Unknown, phosphoenolpyruvate carboxylase domain 0.95 0.99 0.95 1.00 0.30

**EG SYMBOL ACCESSION LOCALIZATION AND FUNCTION GAPs: ALL COC+ THC+ PCP+ CVDC**

*Unknown function, continued*

C6orf114 BC009205 Unknown 0.98 0.98 0.98 0.95 0.31

ZCD1 BC005962 Unknown, mitochondrial 0.07 0.06 0.14 0.04 0.41

C15orf15 BC016725 Unknown, ribosomal 0.05 0.05 0.13 0.00 0.63

C15orf15 BC016777 Unknown, ribosomal 0.06 0.06 0.13 0.03 0.52

C5orf13 BC019068 Unknown 0.04 0.00 0.14 0.01 0.42

C7orf36 BC022043 Unknown 0.07 0.03 0.11 0.03 0.50

C20orf107 BC014951 Unknown 0.95 0.98 0.97 0.99 0.32

C12orf41 BC009746 Unknown 0.94 1.00 0.84 0.96 0.35

FLJ21820 BC017473 Unknown 0.95 1.00 0.86 0.98 0.31

C11orf76 BC004224 Unknown 0.95 0.94 0.96 0.96 0.29

TMEM85 BC002583 Unknown 0.04 0.04 0.03 0.01 0.54

LOC93622 BC021542 Unknown 0.98 0.99 0.96 0.98 0.33

OCIAD1 BC003409 Unknown 0.06 0.06 0.13 0.00 0.55

RBED1 BC010991 Unknown 0.92 1.00 0.74 0.97 0.41

PCID2 BC016614 Unknown 0.94 0.99 0.85 0.97 0.35

MGC10955 BC004960 Unknown 0.95 1.00 0.85 1.00 0.38

MGC13114 BC007207 Unknown 0.95 0.95 0.90 0.99 0.32

MGC16037 BC007651 Unknown 0.93 0.94 0.96 0.93 0.33

FAM113B BC016154 Unknown 0.96 0.98 0.94 0.99 0.46

MGC3771 BC001809 Unknown 0.96 0.96 0.98 0.94 0.27

STK32C BC015792 Unknown 0.93 1.00 0.92 0.99 0.52

WDR62 BC017261 Unknown, WD40 repeat 0.03 0.01 0.10 0.01 0.36

WDR21A BC018979 Unknown, WD40 repeat 0.92 0.99 0.86 0.98 0.52

ZNF625 BC007868 Unknown, RNA binding 0.92 0.96 0.86 1.00 0.35

*Table S2. Full list of significantly regulated transcripts in postmortem aPFC from cocaine, phencyclidine and/or cannabis abusers*. One-hundred and thirty-nine transcripts were significantly regulated by group average *p*-value (GAP) criteria. Overweight/obese controls with cardiovascular disease (CVDC) compared to normal/overweight controls (*Table S1*) illustrated that drug-induced cellular stress was not an underlying cause in the common transcriptional patterns in the drug abuse cases. Gene symbols were annotated using GenBank Accession numbers and EntrezGene.
